# Association Between Assisted Reproductive Technology and White Matter Injury in Premature Infants: A Case-Control Study

**DOI:** 10.3389/fped.2021.686670

**Published:** 2021-08-27

**Authors:** Xuejiao Huang, JianHua Fu

**Affiliations:** Department of Pediatrics, Shengjing Hospital of China Medical University, Shenyang, China

**Keywords:** assisted reproductive technology, brain injury, premature infants, diffusion weighted imaging, white matter injury

## Abstract

**Objectives:** Whether there is a link between assisted reproductive technology (ART) and brain damage in premature infants remains unclear. The aim of this study was to determine whether premature infants conceived by ART are at a greater risk of developing white matter injury (WMI), as detected by magnetic resonance imaging (MRI) or diffusion-weighted imaging (DWI) within 14 days, than those naturally conceived (NC).

**Methods:** A retrospective case-control study was conducted on singleton premature infants with a gestational age of ≥28 weeks and <34 weeks delivered between 2017 and 2019 at Shengjing Hospital, China Medical University. This study included 638 live births that were stratified into case group (*n* = 218) and control group (*n* = 420), depending on the presence or absence of WMI. The exposure proportion of ART was compared between the case and control groups, and a logistic regression model was used to identify whether ART was an independent risk factor for WMI.

**Results:** In the univariate analysis, the exposure proportion of ART conception was higher in cases than in controls (12.84 vs. 7.38%, *p* = 0.024). According to the multivariable analysis, after adjustment for other variables, the association between ART and WMI remained significant (1.82; 95% confidence interval, 1.04–3.21; *P* = 0.038).

**Conclusions:** Singleton premature infants conceived by ART have a higher risk of WMI than NC infants. Given that ART is an independent risk factor for WMI in premature infants, more attention should be paid to neurodevelopmental outcomes in this group.

## Introduction

Since the birth of the first *in vitro* fertilization (IVF)–conceived infant in 1978, assisted reproductive technology (ART) has become one of the primary methods to treat infertility. In recent years, the proportion of newborns conceived by ART has been increasing. In developed countries, the proportion of live births after ART can reach 1.5–5.9% (1, 2). Assisted reproductive technology involves ovulation induction, acquisition and storage of the sperm and ovum, *in vitro* fertilization, intracytoplasmic sperm injection (ICSI), and *in vitro* culture of the fertilized zygote, which may affect the offspring (3). Although the majority of offspring conceived by ART demonstrate comparable health with naturally conceived (NC) children (4), some studies have indicated that ART increases the risk of adverse neonatal outcomes, such as preterm delivery, low birth weight, small-for-gestational-age (SGA), and birth defects (2–6). Furthermore, although previous studies have also reported the effects of ART on offspring growth and metabolic diseases, nervous system development, cognitive and psychosocial development, reproductive function, and cancer risk, they have not reached a unified conclusion (7, 8). It has been suggested that newborns conceived by ART are more likely to have adverse health outcomes than naturally conceived (NC) newborns, probably due to the high proportion of multiple pregnancies caused by ART (9). However, adverse health outcomes have also been reported in singleton births (10).

Prenatal brain development is a highly complex process and can easily be disturbed. It is noteworthy to mention that the manipulation of gametes and embryos during ART exposes them to altered environments during a critical period, which may subsequently impact brain development (11). White matter injury (WMI) is the major form of brain injury among premature infants (12), and some studies have reported that the incidence of WMI in infants with very low birth weight can be as high as 50% (13). Severe WMI can cause periventricular leukomalacia, with sequelae such as cerebral palsy, cognitive and behavioral abnormalities, audiovisual dysfunction, and epilepsy. Of note, some studies have observed that ART is associated with poor neurodevelopment, including cerebral palsy, epilepsy, and intellectual disability (14–16), but almost no studies mention the relationship between ART and WMI. We speculate that WMI is an important mediator between ART and adverse neurodevelopmental outcomes.

Therefore, the aim of this study was to determine the association between the use of ART and the risk of WMI detected by magnetic resonance imaging (MRI) and diffusion-weighted imaging (DWI); this study also aimed to determine whether ART is an independent risk factor for WMI. We hypothesized that ART would be associated with an increased risk of WMI.

## Methods

### Population

This retrospective case-control study was conducted on singleton premature infants treated between January 2017 and December 2019 at the first neonatal intensive care unit of our institution. All singleton premature infants with a gestational age (GA) of ≥28 weeks and <34 weeks who underwent head MRI and DWI within the first 2 weeks after birth were included. Because of the retrospective study design, the clinical data of only preterm infants <34 weeks could be collected for comparison, necessitating the exclusion of late preterm infants from this study. The exclusion criteria were as follows: (a) congenital brain malformations; (b) brain damage due to severe cerebral hemorrhage or inherited metabolic diseases; (c) vanishing twin pregnancies; and (d) incomplete medical records. The infants were stratified into case and control groups, depending on the presence or absence of WMI. The study protocol was approved by the ethical committee of Shengjing Hospital Affiliated to China Medical University (ethical code: 2021PS524K).

### MRI Acquisition and Diagnosis of WMI

All babies relied on natural sleep to cooperate with the MRI and DWI sequence examinations of the head when they were clinically stable. The MRI scan was performed using a Philips Intera Achieva 3.0T superconducting MR instrument, and the scan parameters were as follows: T1WI sequence, TR/TE = 200 ms/2.3 ms, matrix = 224 × 162, field of view = 180 × 150 × 89 mm^3^, section thickness = 5 mm; T2WI: TR/TE = 5,000 ms/80 ms, matrix = 240 × 135, field of view = 180 × 150 × 90 mm^3^, section thickness = 5 mm; DWI: TR/TE = 3,500 ms/63 ms, matrix = 112 × 112, field of view = 180 × 180 × 89 mm^3^, section thickness = 5 mm. Two neuroradiologists who were blinded to the type of conception evaluated all MRI scans.

WMI was diagnosed following Cornette's criteria and divided into punctate white matter injury (PWMI) and diffuse white matter injury (DWMI), based on the MRI and DWI results within the first 2 weeks after birth. PWMI manifests as a point, cluster, and linear DWI high signal at the centrum semiovale or periventricular area, with or without a T1-high signal and T2-low signal. DWMI manifests as varying hyperintensity ranges on DWI in the white matter around the lateral ventricle and involves the deep periventricular, central, and subcortical white matter (17).

### Data Collection

Completed demographic and clinical data were extracted from the medical records. The neonate's sex, GA, birth weight, 1- and 5-min Apgar scores, conception method, patent ductus arteriosus (PDA), and the use of invasive ventilation were recorded. The mother's information included the type of delivery, age, perinatal steroid administration, premature rupture of membranes (PROM), chorioamnionitis, gestational hypertension, and gestational diabetes. ART was defined as infants conceived using standard IVF or ICSI.

### Statistical Analyses

All data were classified as follows: sex (male vs. female), GA (<32 vs. ≥32 weeks), birth weight (<1,500 vs. ≥1,500 g), small-for-gestational age (SGA; Yes vs. No), asphyxia (Yes vs. No), PDA (Yes vs. No), invasive ventilation (Yes vs. No), ART (Yes vs. No), mother's age (<35 vs. ≥35 years), type of delivery (natural or cesarean), prenatal steroid administration (Yes vs. No), PROM (Yes vs. No), chorioamnionitis (Yes vs. No), gestational hypertension (Yes vs. No), and gestational diabetes (Yes vs. No). The data are expressed as the number of cases and the percentage [n (%)], and were compared using the Chi-square test. Univariable logistic regression was conducted to estimate crude association between mother and infant variables and odds of WMI. Variables with *P*-value ≤0.15 were considered as potential significant determinants of WMI and were included in multivariable logistic regression. Multicollinearity between the independent variables was assessed by studying the variance inflation factor. Data were analyzed using the SPSS version 25.0 at 0.05 significance level.

## Results

### Population

**Figure 1:**
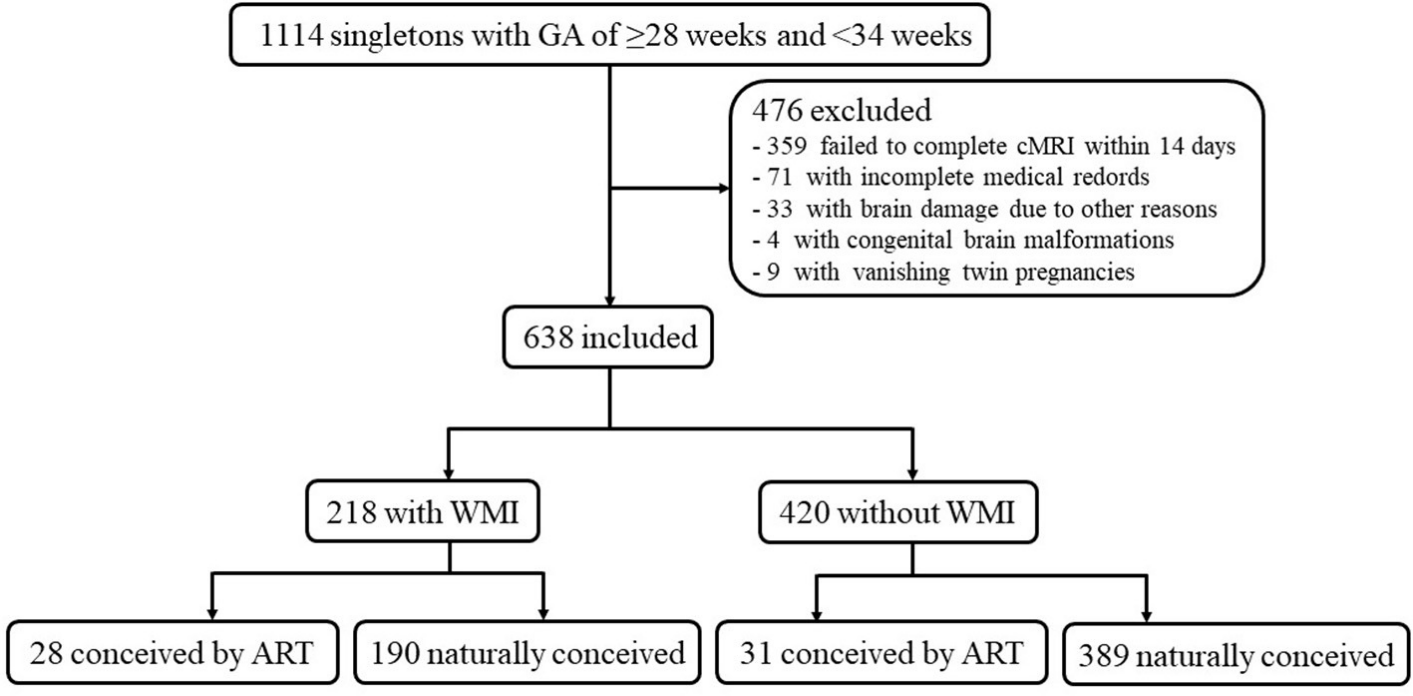
Participant flowchart.

There were 1,114 singleton premature infants with a GA of ≥28 and <34 weeks, of which 476 infants were excluded (Figure 1). These excluded infants comprised the following: (a) 359 infants failed to complete head MRI within 14 days; (b) 71 with incomplete medical records; (c) 33 with severe cerebral hemorrhage or inherited metabolic diseases; (d) nine cases of vanishing twin pregnancies; and (e) four cases with congenital brain malformations. Eventually, a total of 638 patients were available for analysis, which included 218 cases and 420 controls.

### Univariate Analyses of Baseline Variables of Premature Infants in the Case and Control Groups

The infants of the case group were more likely to have asphyxia (31.65 vs. 22.38%, *p* = 0.011) and require invasive ventilation (43.12 vs. 29.29%, *p* < 0.001). The case group had a higher exposure proportion of ART conception (12.84 vs. 7.38%, *p* = 0.024; Table 1).

**Table 1:** Univariate analyses of baseline of premature infants in the case and control groups.

**Variable**	**WMI**		**OR (95% CI)**	***P*-value**
	**No (*n* = 420)**	**Yes (*n* = 218)**		
Sex	
Girl (*n*, %)	170 (40.48)	81 (37.16)	Reference 1.15 (0.82–1.61)	0.416
Boy (*n*, %)	250 (59.52)	137 (62.84)		
GA	
<32 weeks (*n*, %)	137 (32.62)	82 (37.61)	Reference 1.25 (0.89–1.75)	0.208
≥32 weeks (*n*, %)	283 (67.38)	136 (62.39)		
Birth weight	
<1500 g (*n*, %)	76 (18.10)	39 (17.89)	Reference 0.99 (0.64–1.51)	0.949
≥1500 g (*n*, %)	344 (81.90)	179 (82.11)		
SGA	
No (*n*, %)	370 (88.10)	201 (92.20)	Reference 0.63 (0.35–1.11)	0.109
Yes (*n*, %)	50 (11.90)	17 (7.80)		
Asphyxia	
No (*n*, %)	326 (77.62)	149 (68.35)	Reference 1.61 (1.11–2.32)	0.011
Yes (*n*, %)	94 (22.38)	69 (31.65)		
PDA	
No (*n*, %)	347 (82.62)	166 (76.15)	Reference 1.49 (1.00–2.22)	0.051
Yes (*n*, %)	73 (17.38)	52 (23.85)		
Invasive ventilation	
No (*n*, %)	297 (70.71)	124 (56.88)	Reference 1.83 (1.30–2.57)	<0.001
Yes (*n*, %)	123 (29.29)	94 (43.12)		
ART	
No (*n*, %)	389 (92.62)	190 (87.16)	Reference 1.85 (1.08–3.17)	0.024
Yes (*n*, %)	31 (7.38)	28 (12.84)		

*GA, gestational age; SGA, small-for-gestational-age; PDA, patent ductus arteriosus; ART, assisted reproductive technology*.

### Univariate Analyses of Baseline Variables of Mothers in the Case and Control Groups

A greater proportion of mothers in the case group than in the control group had PROM (44.95 vs. 33.10%, *p* = 0.003) and gestational diabetes (29.36 vs. 22.14%, *p* = 0.045); however, a lower proportion of mothers in the case group had cesarean delivery (60.55 vs. 74.76%, *p* < 0.001) and gestational hypertension (20.18 vs. 31.67% *p* = 0.002) than those in the control group (Table 2).

**Table 2:** Univariate analyses of baseline of mothers in the case and control groups.

**Variable**	**WMI**		**OR (95% CI)**	***P*-value**
	**No (*n* = 420)**	**Yes (*n* = 218)**		
Maternal age at child birth				
<35 years	318 (75.71)	157 (72.02)	Reference 1.21 (0.84–1.75)	0.310
≥35 years	102 (24.29)	61 (27.98)		
Type of delivery				
Natural	106 (25.24)	86 (39.45)	Reference 0.52 (0.37–0.74)	<0.001
Cesarean	314 (74.76)	132 (60.55)		
Prenatal steroid administration				
No	163 (38.81)	95 (43.58)	Reference 0.82 (0.59–1.14)	0.244
Yes	257 (61.19)	123 (56.42)		
PROM				
No	281 (66.90)	120 (55.05)	Reference 1.65 (1.18–2.31)	0.003
Yes	139 (33.10)	98 (44.95)		
Chorioamnionitis				
No	353 (84.05)	176 (80.73)	Reference 1.26 (0.82–1.93)	0.292
Yes	67 (15.95)	42 (19.27)		
Gestational hypertension				
No	287 (68.33)	174 (79.82)	Reference 0.55 (0.37–0.81)	0.002
Yes	133 (31.67)	44 (20.18)		
Gestational diabetes				
No	327 (77.86)	154 (70.64)	Reference 1.46 (1.01–2.12)	0.045
Yes	93 (22.14)	64 (29.36)		

*WMI, white matter injury; PROM, premature rupture of membranes*.

### Multivariate Analyses of Mother and Neonate Variables Associated With WMI

After adjusting for the influence of confounding factors, ART conception was identified as an independent risk factor for WMI. After adjusting for the effects of SGA, asphyxia, PDA, invasive ventilation, cesarean, PROM, gestational hypertension, and gestational diabetes, the odds ratio of ART conception was 1.82 (95% confidence interval: 1.04–3.21; Table 3).

**Table 3:** Multivariate analyses of mother and neonate variables associated with WMI.

**Variable**	**OR**	**95%CI**	***P*-value**
SGA	0.68	0.36–1.28	0.231
Asphyxia	1.55	1.03–2.34	0.037
PDA	1.32	0.87–2.02	0.192
Invasive ventilation	1.63	1.11–2.40	0.012
ART-conceived	1.82	1.04–3.21	0.038
Cesarean delivery	0.57	0.39–0.83	0.003
PROM	1.57	1.08–2.28	0.019
Gestational hypertension	0.81	0.51–1.30	0.384
Gestational diabetes	1.29	0.87–1.92	0.201

*SGA, small-for-gestational-age; PDA, patent ductus arteriosus; PROM, premature rupture of membranes*.

## Discussion

ART has been available for 40 years, and people are increasingly concerned about the possible adverse effects of this technology on the health of mothers and babies. Previous studies on the health risks of children conceived through ART have focused mainly on the impact of ART on birth defects, and the research on ART and its neurodevelopmental risk on offspring was insufficient. Previous studies have shown that ART is associated with poor neurodevelopmental outcomes. An Australian cohort study observed an increased risk of cerebral palsy in singletons conceived with ART (14). In addition, studies have observed that children conceived by ART have an increased risk of intellectual disability and epilepsy (15, 16). Cerebral palsy, epilepsy, and cognitive behavioral abnormalities are serious sequelae of WMI; therefore, we speculate that WMI may be an important mediator between ART and poor neurological performance.

To the best of our knowledge, only few previous studies have investigated the impact of ART on WMI in preterm infants. In our study, regardless of whether univariate or multivariate analysis was performed, we found that ART is associated with WMI in preterm infants, confirming that ART is an independent risk factor for WMI in preterm infants. However, we did not follow up these children and, therefore, our study only provides a potential partial explanation of the association between ART and poor neurodevelopmental outcomes.

There are many potential mechanisms by which ART may be related to WMI, such as the effects of fertility hormone therapies used during ART, micromanipulation involved in ART procedures, and prenatal and perinatal complications related to ART treatment. Kalra et al. believe that during the routine ART superovulation stimulation process, the mother's internal environment will change, and that these changes can affect embryo transfer, trophoblast cell implantation, and placenta formation, thereby causing early embryo development retardation (18). The placenta seems to be more vulnerable after ART treatment. Studies have shown that there is a significant increase in the expression of NFκB in ART placental tissue (19), and the upregulation of NFκB has been confirmed to be related to the pathogenesis of WMI (20, 21). In addition, in the critical early period of brain development, fetal steroid hormones are believed to play a role in the epigenetic fetal programming mechanism. In ART, the ovaries can produce estradiol in response to injected gonadotropins, exposing the embryo to supraphysiological levels of estradiol, directly impacting fetal growth, cell differentiation, and gene expression (22, 23). In addition, due to the particularity of intervention methods, ART involves unnatural conception processes such as ovulation induction and gamete manipulation. These manipulations may interfere with epigenetic stability from different aspects (3). Epigenetics is defined as hereditary changes in gene expression without alterations in DNA sequence (24). It has been confirmed in animal models that the expression of miRNAs miR-122, and miR-144 and miR-211, involved in the regulation of neuronal migration and differentiation, were downregulated in the brains of offspring exposed to superovulated environment (22). In addition, one study observed altered DNA methylation patterns at specific genomic loci in bloodspot tests of newborn infants (25). Therefore, the relationship between WMI and ART has a specific pathophysiology, and the specific mechanism underlying this pathophysiology needs further study.

The main advantage of this study is that all research participants were singleton premature infants, which controls the confounding influence of multiple births and preterm birth on WMI to a certain extent. In addition, the case and control groups had different exposures to hypertension and diabetes during pregnancy, but this potential confounding effect was adjusted through multivariable logistic regression.

However, this study has some limitations. Because only fewer babies are conceived with ART, we could not investigate the impact of different kinds of ART on WMI alone. In addition, we could not identify the infants who were conceived only with ovulation induction or intrauterine insemination; therefore, they are included in non-ART. If these treatments are also associated with an increased risk of WMI, our risk estimates may be small. However, there is limited literature in this area. Owing to clinical instability, some infants did not complete the head MRI examination within 2 weeks, which may have introduced selection bias. There remains considerable gaps in the literature on the impact of ART on the neurodevelopment of offspring, and greater work needs to be done in the future. Well-designed prospective studies with large sample size need to be conducted to further verify the conclusions of our study, control for as many potential confounders as possible—such as uterine placental blood circulation, and compare the effects of different ART protocols.

In summary, our study results show that premature infants conceived by ART are at an increased risk of WMI. This finding is relevant for providing appropriate information to parents considering assisted conception. Doctors providing ART need to be informed of the potential risks of the technology so that they can strictly grasp its indications. Neonatologists need to consider the health risks of ART on premature infants and pay attention to their nervous system development outcomes. In addition, this finding needs confirmation in larger studies and further research is necessary to investigate the underlying mechanism.

## Data Availability Statement

The original contributions presented in the study are included in the article/supplementary material, further inquiries can be directed to the corresponding author/s.

## Ethics Statement

The studies involving human participants were reviewed and approved by the ethics committee of Shengjing Hospital Affiliated to China Medical University. Written informed consent from the participants' legal guardian/next of kin was not required to participate in this study in accordance with the national legislation and the institutional requirements.

## Author Contributions

XJH and JHF contributed to the study design. XJH performed the data collection and analysis and drafted the manuscript. JHF reviewed the manuscript. All authors have read and approved the final manuscript.

## Conflict of Interest

The authors declare that the research was conducted in the absence of any commercial or financial relationships that could be construed as a potential conflict of interest.

## Publisher's Note

All claims expressed in this article are solely those of the authors and do not necessarily represent those of their affiliated organizations, or those of the publisher, the editors and the reviewers. Any product that may be evaluated in this article, or claim that may be made by its manufacturer, is not guaranteed or endorsed by the publisher.
